# Denture Induced Submandibular Hematoma in a Patient on Warfarin

**DOI:** 10.1155/2018/4245809

**Published:** 2018-12-17

**Authors:** Yeap Boon Tat, Rajesh Kumar Muniandy, Lily Ng Mooi Hang

**Affiliations:** ^1^Medicine Based Department, Faculty of Medicine and Health Sciences, Universiti Malaysia Sabah, Kota Kinabalu, Sabah, Malaysia; ^2^Department of Anaesthesiology and Critical Care, Queen Elizabeth Hospital, Kota Kinabalu, Sabah, Malaysia

## Abstract

A 79-year-old lady, who was taking warfarin, presented to the Emergency Department with a painless anterior neck swelling, which was associated with hoarseness of voice, odynophagia, and shortness of breath. She first noticed the swelling after she removed her dentures in the evening. On examination, she had an increased respiratory rate. There was a large submandibular swelling at the anterior side of her neck. Upon mouth opening, there was a hematoma at the base of her tongue, which extended to both sides of the tonsillar pillars. The patient was intubated with a video laryngoscope due to her worsening respiratory distress. Intravenous vitamin K and fresh frozen plasma were given immediately. The patient was admitted to the ICU for ventilation and observation. The hematoma subsided after 2 days and she was discharged well.

## 1. Introduction

There are few reports of upper airway hematomas after anticoagulation following minor intraoral trauma. Although some cases can be treated conservatively, others may progress to life-threatening situations. This is an interesting case of a patient with warfarin, who presented with upper airway hematoma after the use of her dentures. The management is also discussed.

## 2. Case Report

A 79-year-old lady presented to the Emergency Department with a painless anterior neck swelling. The swelling appeared one day before and was gradually increasing in size. She first noticed the swelling after she removed her dentures in the evening. Several hours later, she developed hoarseness of voice, odynophagia, and mild shortness of breath. There was no hematemesis, melena, or any neurological deficits. On further questioning, she had a history of hypertension and atrial fibrillation. She has been taking amlodipine and warfarin but was not compliant to her medication and follow-ups. She was supposed to be on warfarin of 3mg per day. However, she did not present herself to the clinic for warfarin optimization, and no INR was done.

On examination, she was fully conscious. However, an audible stridor was heard. Her blood pressure was 130/90mmHg, heart rate 92/min, respiratory rate 24/min, and oxygen saturation 95% on room air. There was a large submandibular swelling at the anterior side of her neck. The mass extended to the left side of neck and was 8cm by 6cm in size. The skin overlying the swelling was bluish-red, and there was tenderness on palpation. Upon mouth opening, there was a hematoma at the base of her tongue which extended to both sides of the tonsillar pillars. The uvula appeared edematous and engorged. There was limitation in mouth opening due to the pain. Bleeding was seen from the base of the tongue when her tongue was protruded.

Blood investigations revealed hemoglobin of 12.4g/dl, platelet 274 x 10^9^/liter, with normal electrolytes. The Prothrombin Time (PT) and Partial Thromboplastin Time (PTT) were prolonged for more than 2 minutes and INR was 8.0. A flexible nasoendoscopy done by the otorhinolaryngologist found a huge swelling at the laryngeal area. The vocal cords were not visible. There were blood clots covering both the arytenoids and epiglottis.

The patient was planned for tracheal intubation due to her worsening respiratory distress. Intravenous vitamin K and fresh frozen plasma (FFP) were given immediately.

Anaesthetic management at that time was gas induction with Sevoflurane in incremental concentration until loss of consciousness with preservation of spontaneous breathing. Muscle relaxants were not used. A D-blade video laryngoscope CMAC was used. During intubation, it was noted that the laryngeal apertures were grossly engorged and swollen. The oral structures were beyond recognition. Blood clots were seen covering the pharyngeal walls, epiglottis, and arytenoids. The vocal cords were not prominently visualized. As the patient was spontaneously ventilated, air bubbles were seen from the posterior side of the engorged epiglottis. This served as a guide for tracheal tube insertion (Video 1). A size 6 endotracheal tube was used to secure the airway. It was a successful intubation only at the second attempt. Hemodynamics were stable throughout the procedure.

The patient was sent to the ICU for ventilation and observation. The hematoma at the neck and base of tongue subsided after 2 days. Her INR was 2.0, and she was discharged from the ICU soon after that.

## 3. Discussion

Anticoagulants are used for prophylaxis and treatment of thromboembolic diseases, acute ischemic strokes, deep venous thrombosis, pulmonary emboli, heart valve diseases, acute myocardium infarction, and atrial fibrillation. Warfarin (brand names Coumadin) is a first generation oral anticoagulant agent. It works by inhibiting the production of vitamin-dependent coagulation factors by the liver [1]. Warfarin needs to be used cautiously due to its narrow therapeutic window and dosing is affected by genetic variation, drug interaction, and nutrition [2].

During the use of warfarin, a significant life-threatening complication is haemorrhage. Although rare, spontaneous upper airway hematoma haemorrhage in patients on anticoagulant therapy of varying severity has been reported [3–9]. Most upper airway hematomas were sublingual hematomas (66.57%) followed by retropharyngeal hematomas (27.03%). Of the cases, 48.65% were reported to be managed conservatively while the rest underwent either cricothyrotomy or tracheal intubation [10]. The complications of upper airway hematomas included respiratory compromise, which was seen in almost half of the cases, followed by pulmonary oedema [11], aspiration pneumonia [8], and mild pneumonitis [12]. There was also a reported case of a patient who died due to anoxic brain injury secondary to upper airway hematoma [13].

When using warfarin, the risk of major bleeding within one year of use ranges between 0.5 to 7.0%, and this risk is directly proportional to the warfarin dosage. This haemorrhagic risk increases in patients whose INR was 6 or more [14] and also in elderly patients who were concurrently on nonsteroid anti-inflammatory and methyl-salicylate.

The commonest presentation of sublingual hematoma is hoarseness of voice and painless swelling in the oral cavity or neck. Physical examination may confirm a sublingual swelling. In our patient, a small laceration at her lower jaw was seen, most probably due to trauma during removal of her dentures. This event initially developed a painless anterior neck swelling, which was ignored by the patient. The traumatic injury in the sublingual area however became worst, causing a retropharyngeal and epiglottic hematoma.

Haemorrhage and hematoma of the oral cavity can be fatal. Bleeding and hematoma into the sublingual and submaxillary spaces may also create a pseudo-Ludwig's phenomenon [5]. With an acute expanding hematoma, the tongue and floor of the mouth may become elevated. This will lead to an airway obstruction.

If diagnosed early and managed promptly, the prognosis will be good, and the haematomas may resolve completely. Once diagnosed, these patients will require prompt airway management, reversal of anticoagulation, as well as close monitoring. Airway management should be performed ideally with awake intubation, while maintaining a spontaneous respiration. However, in a situation where tracheal intubation is not successful, emergency cricothyroidotomy or surgical tracheostomy should be performed for definitive airway stabilization.

In severe sublingual hematoma, anticoagulation should be reversed immediately with Prothrombin Complex Concentrate (PCC) or Fresh Frozen Plasma (FFP), followed by vitamin K. Both oral and intravenous routes for vitamin K sufficiently reverse anticoagulation and result in similar reductions in INR at 24 hours [15]. Although both PCC and FFP can be used, PCC has several advantages over FFP. PCCs have smaller infusion volumes and have an enhanced safety because of viral inactivation [16]. In the treatment of our case, oral warfarin was discontinued and FFP 15ml/kg was given. PCC was not available at our centre.

## 4. Conclusion

Warfarin-induced submandibular hematoma can cause airway obstruction. In these patients, early definitive airway management is crucial. A rapid medical management is also crucial to reverse the effects of warfarin.

## Data Availability

No data were used to support this study.

## Supplementary Materials

Supplementary Materials Video 1: air bubbles seen from the posterior side of the engorged epiglottis, which served as a guide for tracheal tube insertion.
